# Serological detection of Newcastle disease virus in backyard poultry production system in Woliso District South West Shewa, Central Ethiopia

**DOI:** 10.1002/iid3.1179

**Published:** 2024-02-01

**Authors:** Alemayehu Choramo, Motuma Debelo, Asamenew Tesfaye, Chala Bedasa, Chala Dima, Chala Guyassa

**Affiliations:** ^1^ Department of Veterinary Medicine Bonga University College of Agriculture and Natural Resource Bonga Ethiopia; ^2^ School of Veterinary Medicine Jimma University College of Agriculture and Veterinary Medicine Jimma Ethiopia; ^3^ Viral Serology Division National Animal Health Diagnosis and Investigation Center Sebeta Ethiopia

**Keywords:** backyard‐poultry, detection, serology, Woliso

## Abstract

**Background:**

Newcastle disease (ND) is one of the most important respiratory viral diseases. The disease is endemic in many parts of Ethiopia. However, there is no clear record about the introduction of the virus to the country (Ethiopia). Hence, detail about the ND is very important in its (ND) control and prevention. Despite these facts, there is no available research work done on ND in the current research area that would help either as references for researchers or that could help in the control and prevention of the disease. Therefore, the objective of this study was to detect the ND virus (NDV), using serological methods in from December 2018 to November 2019.

**Methodology:**

A cross‐sectional type of study was conducted to detect the NDV. The convenience sampling method was used for sample data collection. Before data collection, chicken with previous history of vaccination against the NDV was excluded from the sampling animals. Then, a total of 348 blood samples of 2 mL were collected from the brachial vein in 3 mL disposable syringes. The serum was collected in labeled 2 mL cryovial tubes. Indirect enzyme‐linked immunosorbent assay (ELISA) tests were performed to detect antibodies against NDV and to determine its antibody titer. The test was performed using (ID.vet innovative version 2) procedure.

**Result:**

In the indirect ELISA test, 37.64% (131/348) were positive and antibody titer mean value of (1761.9088) was scored. The standard deviation of 2592.42160 and a percentage coefficient of variation of 147% was scored.

**Conclusion:**

From the finding, we conclude that indirect ELISA test detected the presence of the NDV in the study area and the heterogeneousity of antibody titer in the study area. Therefore, further molecular characterization and epidemiological investigation should be carried and vaccination of animals is critical in the study area.

## Graphical abstract

Cross‐sectional type of study was conducted with the aim of detecting Newcastle disease virus (NDV). Convenience sampling method was used for sample data collection and a total of 348 serum samples were collected and indirect enzyme‐linked immunosorbent assay (ELISA) tests were performed to detect antibodies against NDV and to determine its antibody titer. In the indirect ELISA test, 37.64% (131/348) were positive and antibody titer mean value of (1761.9088) was scored.

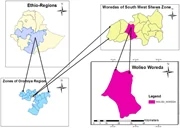

## 1 INTRODUCTION

Poultry industry has been one of the most dynamic and ever‐expanding sectors, contributing much to the global economy. Poultry sector plays a key role in poverty reduction at national and household levels in developing countries.^1^ Poultry meat and eggs are essential sources of nutrition and an important source of income for poor families, and therefore, important for rural development.^2^ Poverty reduction and sustainable development of many developing countries rely on agriculture. Among the agricultural sectors, livestock sector is an important sector, contributing about 47% of the agricultural gross domestic production (GDP) and 18.8% of the national GDP of Ethiopia.^1^

Poultry is one of the essential livestock sectors, which is an important tool to respond rapidly to poverty combating, if included in rural development strategies. Poultry can play important roles in poverty alleviation, nutrition, and food security.^1^ The income from poultry can help the family with everyday needs like payment for education, health care, and clothes. Family poultry can also be the first step to get other livestock like sheep, goats, and cows.^3^

In Ethiopia conditions, chicken production under backyard system has long been practiced in Ethiopia and almost every rural family owns poultry which has been widely used for egg, meat production, and other purposes.^4^ Village chickens contribute more than 98% of the total poultry meat and egg production in the country.^5^ The total chicken population in Ethiopia is estimated to be 56.06 million out of which 97% is an indigenous breeds that are well adapted to the local environmental conditions.^6^ The majority (97%) of these chickens are maintained under this scavenging production system. However, in research extension and development agenda, the village indigenous chickens are poorly considered, focusing the commercial poultry sector which covers only approximately 3%.^7^

Despite its role in raising income and reducing poverty in local communities of Ethiopia, backyard poultry production is hampered by wide arrays of constraints, such as predators, poor management and nutrition,^8^ and infectious diseases (such as Newcastle disease [ND], infectious bursal disease, mycoplasmosis, pasteurellosis and salmonellosis, coccidiosis, and fowl pox) are the major causes of morbidity and mortality in poultry in Ethiopia.^9^

Among the poultry diseases, ND is one of the most important viral diseases. It is an acute infectious viral disease of domestic poultry and other species of birds regardless of difference in sex and age.^10^ The disease is characterized by respiratory problems, nervous system impairment, gastrointestinal, and reproductive problems.^11^

The disease is endemic in different parts of Ethiopia. However, there is no clear record about the introduction of the virus to the country (Ethiopia). Some reports that ND first occurred in and around the seaports of the country and spread to the interior of the country along transport routes. The first documented outbreak of ND in Ethiopia dates to 1971 and reported from a small poultry farm in Asmara, Eritrea, located close to a seaport and the province of the country (former Ethiopia).^12^

Nowadays, the intensification of agriculture is accelerating from time to time in many countries of Africa due to the improvement of novel technologies, financial initiatives, changing social infrastructure, and private sector engagement,^13^ and policies allowing increases in consumption and productivity for a variety of livestock species. Similarly, Ethiopian entrepreneurs are setting up large, intensively managed flocks of exotic breeds, particularly, in areas close to Addis Ababa.^14^ Additionally, government‐owned poultry multiplication centers throughout the country, nongovernmental organizations, and private individuals also distribute intensively reared chickens to smallholders. As a result, more urban and suburban households now keep flocks of 50–1000 birds under semi‐intensive management.^15^ The close links between intensive and smallholder farms could facilitate the spread of diseases like ND and other contagious diseases, exacerbated by low biosecurity, and poor access to veterinary inputs and expertise among smallholder producers.^16^

The district (Woliso) is in close contact through live poultry marketing with Addis Ababa (114 km), which favors the flow of chicken to and from Addis Ababa. The movement of chicken marketing is from periphery to the center (rural to town) and cross‐breed chicken multiplied in commercial farms around Addis Ababa, disseminated away from Addis Ababa,^14^ which enhances the spread of poultry diseases either to Woliso or all over the country. Such movements of chicken give a high chance for spreading of diseases like ND.^17^

ND is a main constraint to the poultry production system in globe as well as in Africa. The village chicken population in many parts of Ethiopia is endemically infected with ND virus (NDV).^18^ Due attention is needed to control and prevent the disease as well as to combat economic loss occurrence due to the disease.^19^ Despite these facts, no researcher or no concerning institution has given attention to ND in the current study area. In Ethiopia, researchers as well as research institutes and academic institutes as focus on East Shewa zone including its surroundings and only few research works from northern parts of Ethiopia. Until this research work, there is no available research work done on ND in the current research area, that would help either as references for researchers or in the control and prevention of the disease.

Therefore, objectives this study was to detection of NDV in backyard poultry production system and to determine the antibody titer against NDV in chicken sera.

## 2 MATERIALS AND METHODS

### 2.1 Study area

**Figure 1:**
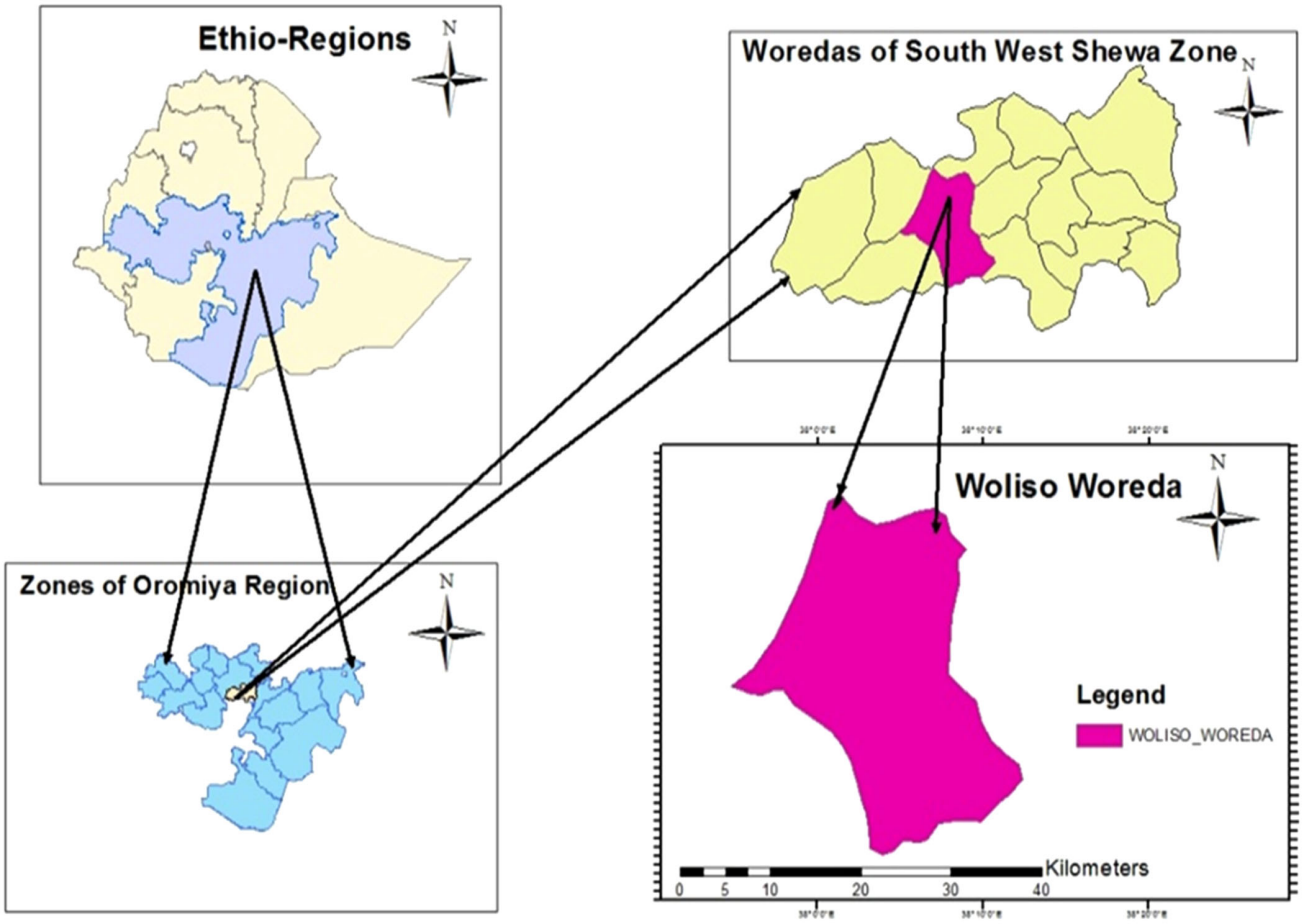
Map of the study area (created by geographic information system software).

The study was conducted in Woliso district, South West Shewa, Oromia Regional State, Central, Ethiopia, Woliso is 114 km away from the capital city Addis Ababa (Figure 1). It geographically lies between longitude of 37°58′16.3″E and latitude of 8°32′23.0″N and 1500–2900 m above sea level. The area is characterized by binomial rainfall, long seasons (from June to September), and short seasons (March to April). The minimum annual temperature is 13.6°C and maximum annual temperature is 25°C with average annual temperature 19.3°C. The livestock population of the district is 224,334 cattle, 39,543 sheep, 51,042 goats, 7625 horses, 6164 mules, 16,320 donkeys, and 147,679 poultry.

### 2.2 Study animals

The study population includes chickens of all ages, both sexes, both cross and local breeds that were managed under the backyard production system and chickens that have no history of vaccination against ND. Before sample collection, owners asked for the vaccination history of his/her chicken. Any chicken with previous history of vaccination against NDV were excluded from sampling.

### 2.3 Study design

A cross‐sectional type of study was carried out from December 2018 to November 2019 to detect antibodies to the NDV in a backyard poultry production system. The convenience sampling method was used to collect sample data for this study. A total 348 blood samples were collected from six kebeles in the district. The collected samples were used to detect antibodies against NDV and to estimate antibody titer.

### 2.4 Sample collection

For the enzyme‐linked immunosorbent assay (ELISA) test, 348 blood samples of 2 mL was collected from the brachial vein in 3 mL disposable syringes, left horizontally for 3 h, and then vertically for the serum to ooze out. Serum was collected in labeled 2 mL cryovial tubes and kept cool in an icebox during collection and transportation to National Animal Health Diagnostic and Investigation Center, Sebata. The serum in the cryovial tubes was stored at −20°C until processing.

### 2.5 Laboratory analysis

An Indirect ELISA technique (ID.vet innovative version 2) kit was used to detect the presence of anti‐NDV antibodies and to determine antibody titer levels in the chicken serum following the kit manufacturers' recommended protocol. Briefly, the test sera were prediluted by dilution buffer 14 in a predilution plate according to the established protocol or kit instructions, and each was dispensed into microwells. In the ELISA plate, prediluted samples and dilution buffer 14 were added and incubated for 30 min ± 3 min at 21°C. After incubation, the sera were discarded from the plates, and each well was washed three times by 300 μL of washing solution. Next, 100 μL anti‐chicken immunoglobulin's peroxidase conjugate was dispensed into the wells and the plates were incubated for 30 min ±3 min at 21°C. After incubation, again, the content was discarded from the plates, and each well was washed three times by 300 μL of washing solution. Then, 100 μL substrate solutions were dispensed into each test well and again incubated for 15 min ± 2 min at 21°C in the dark place. After a final incubation, the substrate chromogen reaction was stopped by adding 100 μL stop solution and the color reactions were quantified by measuring the optical density (OD) of each well at 450 nm using ELISA reader. The test is valid when the mean OD value of the positive control serum is greater than 0.250, and the ratio of the mean value of the positive and negative control (OD_PC_ and OD_NC_) is greater than 3.

Serum sample to positive (SP) control ratio was calculated using the formula below:

$$\mathrm{SP}=\frac{{\mathrm{OD}}_{\text{sample}}-{\mathrm{OD}}_{\text{NC}}}{{\mathrm{OD}}_{\text{PC}}-{\mathrm{OD}}_{\text{NC}}}.$$

If SP value was ≥0.3, the NCD antibody status was considered positive and if SP value < .3, it was taken as negative.

The titer of the antibody in the serum sample of chicken was determined using

$${\mathrm{Log}}_{10}\left(\text{titer}\right)=1.00\times \log_{10}\left(\mathrm{SP}\right)+3.520,$$

$$\text{Titer}=10_{10}^{\log}(\text{tire}).$$

Antibody titer results are interpreted as: If the titer ≤993, it was considered negative, and if the titer ≥993 it was considered positive (ID.vet innovative version 2).

### 2.6 Data analysis

The data collected for serology was entered into Microsoft Ex‐Cell spreadsheet, edited, coded, transferred to SPSS software (version 21), and analyzed by using descriptive statistics. Mean of antibody titer between villages (kebeles), standard deviation, percentage coefficient of variance, and statistical significance (*p* value) was computed. For mean comparison, one‐way analysis method was used. The differences were considered statistically significant (*p* < .05) at 95% confidence interval (CI).

## 3 RESULTS

Out of 348 serum samples tested, 131 serum (37.64%) were positive for antibodies against NDV. The range of the antibody titer was from 0 to 11735.9 in microliter of serum sample. The highest (2308.3) and lowest (1225.7) antibody titer was recorded in Obbi–Koji and Gurura–Baka kebeles, respectively. The mean and standard deviation for the antibody titer was accounted, 1761.9 and 2592.4, respectively, in microliters of serum samples (Table 1). The mean antibody titer was significantly different (*F* = 1.993, *p* = .0079) (Table 1) among the kebeles where the samples were collected. The percentage coefficient of variation (%CV) from the analysis was 147% and this shows that the variation of antibody is very high, indicating the heterogeneity of antibody titer level (Table 1).

**Table 1:** The level of antibody titer and comparison of mean antibody titers using ANOVA.

Study areas	N	Mean	Std. deviation	Std. error	95% confidence interval for mean		Minimum	Maximum	*F* test	*p* Value	%CV
					Lower bound	Upper bound					
1. B/Koricha	56	1320.7	2335.3	312.1	695.2	1946.1	0	8311.52			
2. O/Koji	60	2308.3	2674.6	345.3	1617.3	2999.3	0	10,033.67	1.993	0.0079	147
3. D/Duleti	64	1478.5	2611.6	326.5	826.2	2130.9	0	11,735.9			
4. G/Baka.	58	1225.7	1998.5	262.4	700.3	1751.2	0	7472.78			
5. T/Anchabi	51	2131.4	2762.1	386.8	1354.6	2908.3	0	11,341.63			
6. F/Gora	59	2140	2951.3	384.2	1370.9	2909.2	0	10,358.96			
Total	348	1761.9	2592.4	138.9	1488.6	2035.2	0	11,735.9			

Abbreviations: %CV, percentage coefficient of variation; ANOVA, analysis of variance; B/Koricha, Badessa Koricha; D/Duleti, DireDuleti; F/Gora, .Fodu Gora; G/Baka, GururaBaka; O/Koji, bbi Koji; T/Anchabi, Tombe Anchabi.

## 4 DISCUSSION

The finding in this study is higher than the previous finding of Terefe et al.,^20^ who reported (11.61%) from three Rift Valley districts, namely, Bishoftu, Tikur wuha, and Ziway. This low value could be related to the sampling duration as they collected the sample only within 1 month. In this study, the sample was collected for 11 months from December to November. Collecting samples for a long duration gives a chance to get the pathogen at different infection stages and thus increases the precision of finding. This means, the presence or absence of detectable antibody levels within a flock depends also on the phase of infection at the time of sampling according to Bell and Mouloudi.^21^ Also, other low serological findings reported from Bale Zone (27.86%) and East Shewa Zone (28.6%) of Oromiya region in Ethiopia by Minda et al.^22^ and Desalegn,^23^ respectively. Their lower finding could be attributed to the different serological assays used. The assay they used was hemagglutination (HA) test and hemagglutination Inhibition (HI) test, respectively, but in this research the indirect ELISA test, which is considered as accurate, rapid, and sensitive compared to the HA test and HI test^24^ was used. Also, in the HI test, only the antibodies directed against the HN protein are detected, surprisingly, the ELISA platforms utilizing whole virus as antigens can potentially detect antibodies directed against all proteins in the NDV particle.^25^ However, this serological finding is lower than the findings of Biswas et al.,^26^ who reported 89% again from Bangladesh and Chaka et al.,^19^ who reported 82.6% and 78.6%, respectively, from East Shewa Zone, Ethiopia. This difference might be attributed to the different criteria of ELISA result interpretation methods used. They calculated percentage inhibition (PI) and classified as positive if one or more chickens in the flock tested positive (PI > 40), but we calculated the serum SP control ratio and used different criteria of interpretation as presented in Section 2.5 above. Finally, the current serological finding is in line with previous findings of Zeleke et al.,^27^ who reported (35.9%) from Alage and again Tadesse et al.^28^ finding, who reported (38%) from Adama, Emilia et al.^29^ reported (38.11%) and Parvin et al.,^30^ reported (40%) from Bangladesh. This agreement of our finding to the previous research reported by scholar may be attributed to sampling duration, sampling time (clinical stage), and serological assays used as stated by Bell and Mouloudi.^21^

For indirect ELISA test positives, the antibody titer ranges from 998.01 to 11735.9 with a standard deviation of (2592.42160), this is very high. According to Chaka et al.^9^ report, this wide range of antibody titer levels shows the presence of both class I (responsible for lower level antibody titer) and class II (responsible for higher level antibody titer) NDV. The mean antibody titer of whole kebeles' was 1761.9088 ranging from 1225.7 to 2035.2352 (Table 1) at 95% CI and 5% precision. Our mean value of antibody titer finding is lower than Parvin et al.^30^ report, who reported an antibody titer mean value of 6291 from Bangladesh, this variation of mean titer is may be due to the different test kits used (they used rapid NDV antigen test kit, but we used innovative diagnostic indirect ELISA kit) and higher antibody titer may be attributed to presence of velogenic strains of the virus dominating in their research area, which are known to produce higher antibody titers than lentogenic and mesogenic strains.^11^ Lower mean antibody titer was reported (0–969) from Mozambique by Frechaut et al.^31^ The lower mean values might be due to the reason that the age groups of the participant chicken (in their study, only chickens of 30–40 day old participate) or virus circulation increased and immunity of the flock decreased as antibody titer and virus circulation inversely proportionate (the chickens did not develop sufficiently protective immunity) as stated by Chaka et al.^19^

It could be seen from (Table 1) above that the mean antibody titer of each kebeles shows large values. The standard deviation of antibody of chickens both between groups (kebeles) and within groups (kebeles) shows great variation at 95% CI, which shows the natural exposure of the chickens to the virus, because in the case of vaccination (experimental case) the standard deviation could not be such a large value (Frechaut et al.^31^ and Tesfaye et al.^32^). Moreover, the %CV computed was 147%. The CV is used mostly to evaluate the effectiveness of vaccine programs and the development poultry humoral immune response. According to the previous report of Frechaut et al. a good coefficient of variations (CVs), ranges between 30% and 50%, indicating that flock immunization was achieved by generating homogenous antibody titers.^31^ The higher score of %CV in this study shows that the variation of antibody is very high, indicating the heterogeneity of antibody titer level and natural infection. In case of vaccination, it would be homogenous with %CV less than 30 (strong) and between 30 and 50 (good).^32^ The highest CV in this finding might be due to the natural infections acquired by chickens that provoke immunity.

## 5 CONCLUSION AND RECOMMANDATIONS

The study was conducted to detect NDV and to determine antibody titers using indirect ELISA test methods. Indirect ELISA test revealed that 37.64% chickens were positive for antibodies against NDV. This indicated that the virus was circulating in the study area. The mean antibody titration was found 1761.9088 in microliters of serum samples. The higher standard deviation (2592.42160) and higher %CV (147%) showed the existence of natural exposure to NDV.

Based on the above conclusions, the following recommendations forwarded:

Further molecular characterization should be done to identify genotypes circulating in the area. Epidemiological investigation should be carried out to distinguish the associated risk factors for ND in the study area. Vaccine programs should be should be encouraged to prevent ND outbreak and to minimize economic loss due to high mortality and morbidity of the disease.

## AUTHOR CONTRIBUTIONS

**Alemayehu Choramo**: Data collection; conceptualization; methodology; draft writing and revising. **Motuma Debelo**: Conceptualization; supervision and revising. **Asamenew Tesfaye**: Conceptualisation; methodology and supervision. **Chala Bedasa**: Data collection. **Chala Dima**: Data collection and investigation. **Chala Guyassa**: Validation and laboratory analysis.

## CONFLICT OF INTEREST STATEMENT

The authors declare no conflict of interest.

## ETHICS STATEMENT

Before conducting this research, all the farmers/owners of chicken were informed about the purpose of the study and also they were aware of the importance and benefit of the research in terms of immediate and future values. Besides, the research is highly participatory in the sense that chicken owners provided their chicken as research grounds. Furthermore, while collecting samples, safe handling procedures were followed. A permission to conduct this research was obtained from the Research Ethical Committee and the letter of clearance was received from Jimma University College of Agriculture and Veterinary Medicine. For notification, formal letters were written and sent to the district by the district Agricultural Office.

## Data Availability

The blood serum data used to support the findings of this study are available from the corresponding author upon request.

## ANNEX A TEST PROCEDURE FOR INDIRECT ELISA

Allow all reagents to come to room temperature (21 ± 5°C) before use. Homogenize all reagents by inversion or vortex.

The negative and positive controls are supplied ready to use. Do not add dilution buffer to the control wells A1, B1, C1, and D1‐controls tested with undiluted.

Samples, however, are tested at a final dilution of 1:100 in dilution buffer 14 (1:50 predilution, followed by 1:2 in the microplate).

1. In a predilution plate, aside A1, B1, C1, and D1 for control, and add:
  – 5 µL each sample to be tested.
  – 245 µL Dilution Buffer14 to all well EXCEPT control well A1, B1, C1, and D1.
  *Note*: it is recommended to respect the indicated order of deposit to be able to visually control the addition of samples each well.
2. In the enzyme‐linked immunosorbent assay microplate, add:
  – 100 µL of the negative control on wells A1 and B1.
  – 100 µL of positive control on wells C1 and D1.
  – 50 µL of dilution buffer 14 to as many wells as there are samples to be tested (NOT to control A1, B1, C1, and D1).
  – 50 µL of the prediluted samples as prepared above.
3. Cover the plate and incubate for 30 min ± 3 min at 21°C (±5°C).
4. Prepare the conjugate 1× by diluting the concentrated conjugate 10× to 1:10 in dilution buffer 3.
5. Empty wells. Wash each well 3 times with at least 300 µL of wash solution 1×. Avoid drying of the wells between washings.
6. Add 100 µL of the conjugate 1× to each well.
7. Cover the plate and incubate 30 min ± 3 min at 21°C (±5°C).
8. Empty wells. Wash each well three times with at least 300 µL of wash solution 1×. Avoid drying of the wells between washings.
9. Add 100 µL of the substrate solution to each well.
10. Cover the plate and incubate 15 min ± 2 min at 21°C (±5°C).
11. Add 100 µL of stop solution to each well in the same order as in step No. 9, to stop the reaction.
12. Read and record the optical density at 450 nm (ID.vet innovative Indirect version 2).
